# Supplementary material for: Knockout of Babesia bovis rad51 ortholog and its complementation by expression from the BbACc3 artificial chromosome platform
Source: PLoS One. 2019 Aug 6;14(8):e0215882. doi: 10.1371/journal.pone.0215882 (PMC6684078; doi:10.1371/journal.pone.0215882)
Supplement: S1 Fig — Those proteins for which there is experimental support for catalysis of canonical Rad51 functions are indicated in blue in Fig 1. The Walker A and Walker B motifs are indicated here by red and blue box overlays, respectively. This alignment provided the basis for the phylogenetic tree shown in Fig 1A. (PDF) [file pone.0215882.s001.pdf]

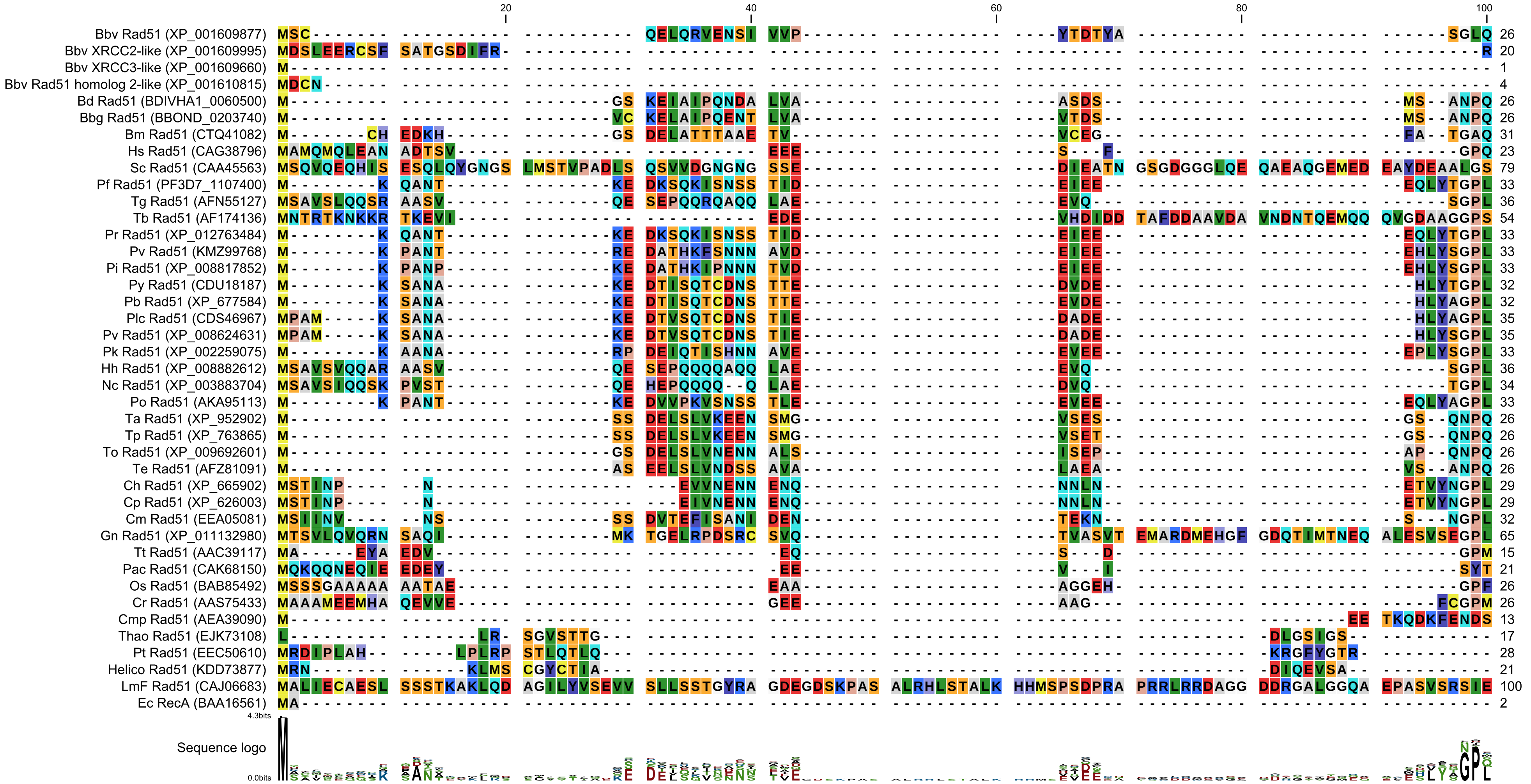
20
40
60
80
100
Bbv Rad51 (XP_001609877) 26
Bbv XRCC2-like (XP_001609995) 20
Bbv XRCC3-like (XP_001609660) 1
Bbv Rad51 homolog 2-like (XP_001610815) 4
Bd Rad51 (BDIVHA1_0060500) 26
Bbg Rad51 (BBOND_0203740) 26
Bm Rad51 (CTQ41082) 31
Hs Rad51 (CAG38796) 23
Sc Rad51 (CAA45563) 79
Pf Rad51 (PF3D7_1107400) 33
Tg Rad51 (AFN55127) 36
Tb Rad51 (AF174136) 54
Pr Rad51 (XP_012763484) 33
Pv Rad51 (KMZ99768) 33
Pi Rad51 (XP_008817852) 33
Py Rad51 (CDU18187) 32
Pb Rad51 (XP_677584) 32
Plc Rad51 (CDS46967) 35
Pv Rad51 (XP_008624631) 35
Pk Rad51 (XP_002259075) 33
Hh Rad51 (XP_008882612) 36
Nc Rad51 (XP_003883704) 34
Po Rad51 (AKA95113) 33
Ta Rad51 (XP_952902) 26
Tp Rad51 (XP_763865) 26
To Rad51 (XP_009692601) 26
Te Rad51 (AFZ81091) 26
Ch Rad51 (XP_665902) 29
Cp Rad51 (XP_626003) 29
Cm Rad51 (EEA05081) 32
Gn Rad51 (XP_011132980) 65
Tt Rad51 (AAC39117) 15
Pac Rad51 (CAK68150) 21
Os Rad51 (BAB85492) 26
Cr Rad51 (AAS75433) 26
Cmp Rad51 (AEA39090) 13
Thao Rad51 (EJK73108) 17
Pt Rad51 (EEC50610) 28
Helico Rad51 (KDD73877) 21
LmF Rad51 (CAJ06683) 100
Ec RecA (BAA16561) 2
4.3bits
Sequence logo
0.0bits

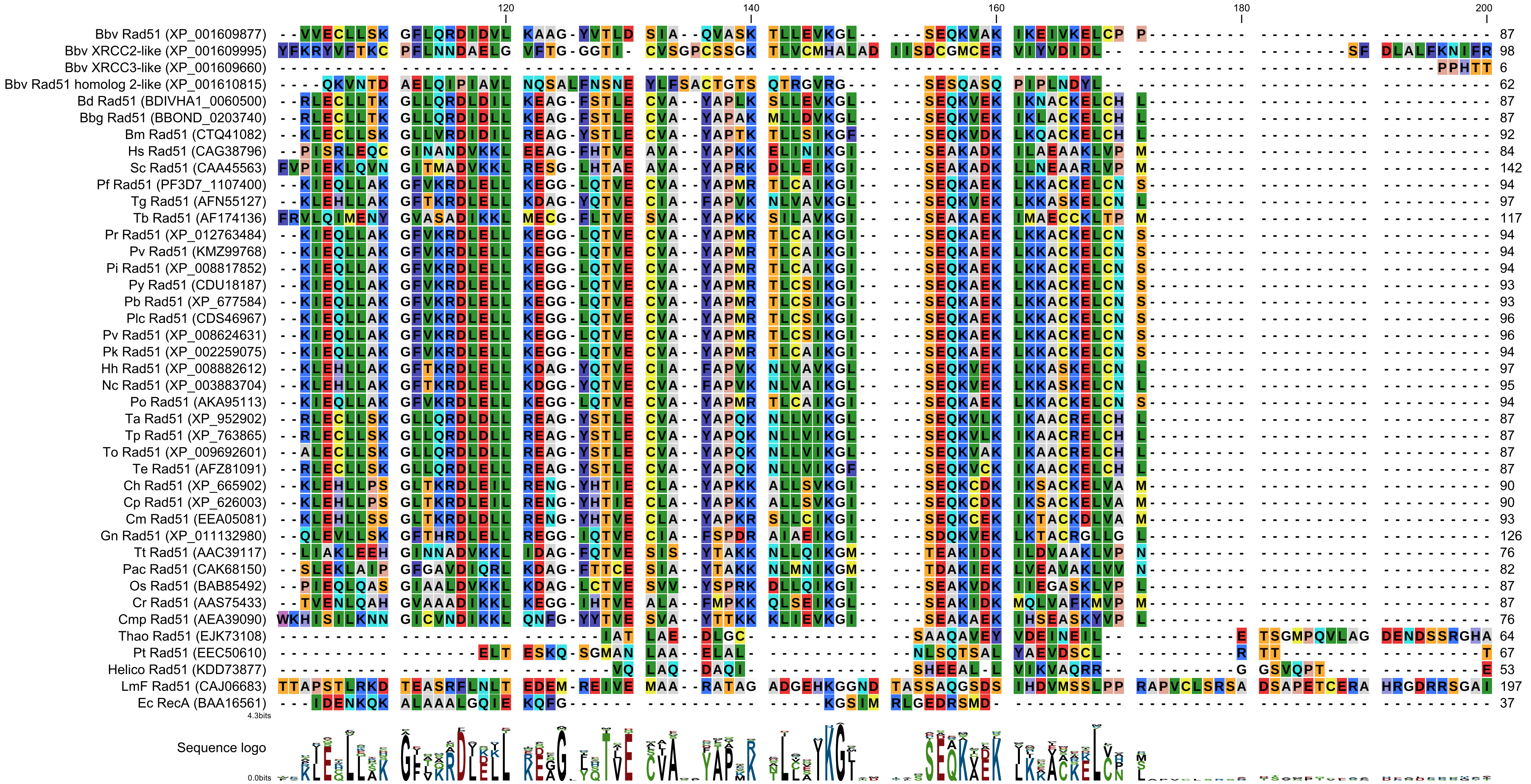
120
140
160
180
200
Bbv Rad51 (XP_001609877) 87
Bbv XRCC2-like (XP_001609995) 98
Bbv XRCC3-like (XP_001609660) 6
Bbv Rad51 homolog 2-like (XP_001610815) 62
Bd Rad51 (BDIVHA1_0060500) 87
Bbg Rad51 (BBOND_0203740) 87
Bm Rad51 (CTQ41082) 92
Hs Rad51 (CAG38796) 84
Sc Rad51 (CAA45563) 142
Pf Rad51 (PF3D7_1107400) 94
Tg Rad51 (AFN55127) 97
Tb Rad51 (AF174136) 117
Pr Rad51 (XP_012763484) 94
Pv Rad51 (KMZ99768) 94
Pi Rad51 (XP_008817852) 94
Py Rad51 (CDU18187) 93
Pb Rad51 (XP_677584) 93
Plc Rad51 (CDS46967) 96
Pv Rad51 (XP_008624631) 96
Pk Rad51 (XP_002259075) 94
Hh Rad51 (XP_008882612) 97
Nc Rad51 (XP_003883704) 95
Po Rad51 (AKA95113) 94
Ta Rad51 (XP_952902) 87
Tp Rad51 (XP_763865) 87
To Rad51 (XP_009692601) 87
Te Rad51 (AFZ81091) 87
Ch Rad51 (XP_665902) 90
Cp Rad51 (XP_626003) 90
Cm Rad51 (EEA05081) 93
Gn Rad51 (XP_011132980) 126
Tt Rad51 (AAC39117) 76
Pac Rad51 (CAK68150) 82
Os Rad51 (BAB85492) 87
Cr Rad51 (AAS75433) 87
Cmp Rad51 (AEA39090) 76
Thao Rad51 (EJK73108) 64
Pt Rad51 (EEC50610) 67
Helico Rad51 (KDD73877) 53
LmF Rad51 (CAJ06683) 197
Ec RecA (BAA16561) 37
4.3bits
Sequence logo
0.0bits

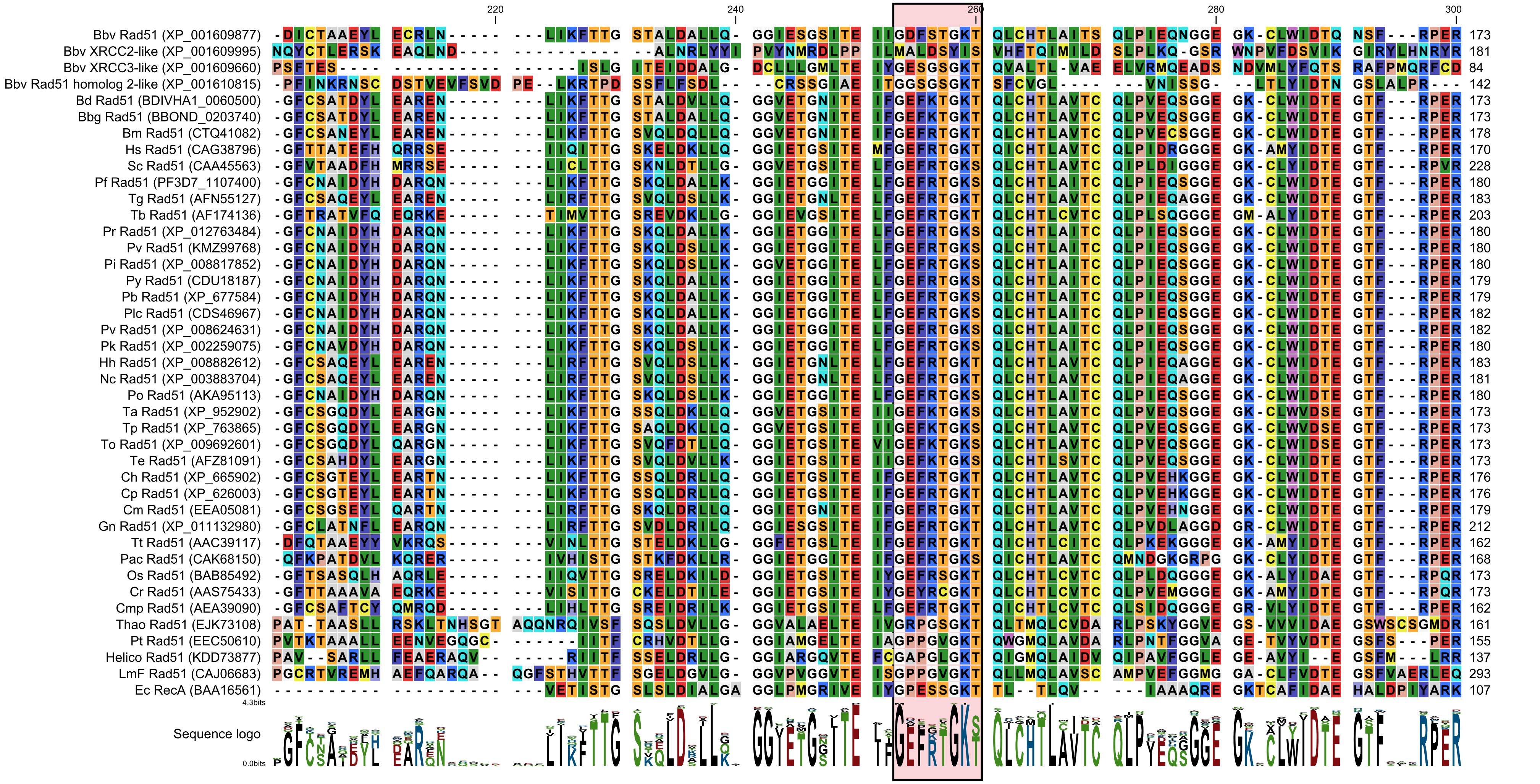
220
240
260
280
300
Bbv Rad51 (XP_001609877) 173
Bbv XRCC2-like (XP_001609995) 181
Bbv XRCC3-like (XP_001609660) 84
Bbv Rad51 homolog 2-like (XP_001610815) 142
Bd Rad51 (BDIVHA1_0060500) 173
Bbg Rad51 (BBOND_0203740) 173
Bm Rad51 (CTQ41082) 178
Hs Rad51 (CAG38796) 170
Sc Rad51 (CAA45563) 228
Pf Rad51 (PF3D7_1107400) 180
Tg Rad51 (AFN55127) 183
Tb Rad51 (AF174136) 203
Pr Rad51 (XP_012763484) 180
Pv Rad51 (KMZ99768) 180
Pi Rad51 (XP_008817852) 180
Py Rad51 (CDU18187) 179
Pb Rad51 (XP_677584) 179
Plc Rad51 (CDS46967) 182
Pv Rad51 (XP_008624631) 182
Pk Rad51 (XP_002259075) 180
Hh Rad51 (XP_008882612) 183
Nc Rad51 (XP_003883704) 181
Po Rad51 (AKA95113) 180
Ta Rad51 (XP_952902) 173
Tp Rad51 (XP_763865) 173
To Rad51 (XP_009692601) 173
Te Rad51 (AFZ81091) 173
Ch Rad51 (XP_665902) 176
Cp Rad51 (XP_626003) 176
Cm Rad51 (EEA05081) 179
Gn Rad51 (XP_011132980) 212
Tt Rad51 (AAC39117) 162
Pac Rad51 (CAK68150) 168
Os Rad51 (BAB85492) 173
Cr Rad51 (AAS75433) 173
Cmp Rad51 (AEA39090) 162
Thao Rad51 (EJK73108) 161
Pt Rad51 (EEC50610) 155
Helico Rad51 (KDD73877) 137
LmF Rad51 (CAJ06683) 293
Ec RecA (BAA16561) 107
4.3bits
Sequence logo
0.0bits

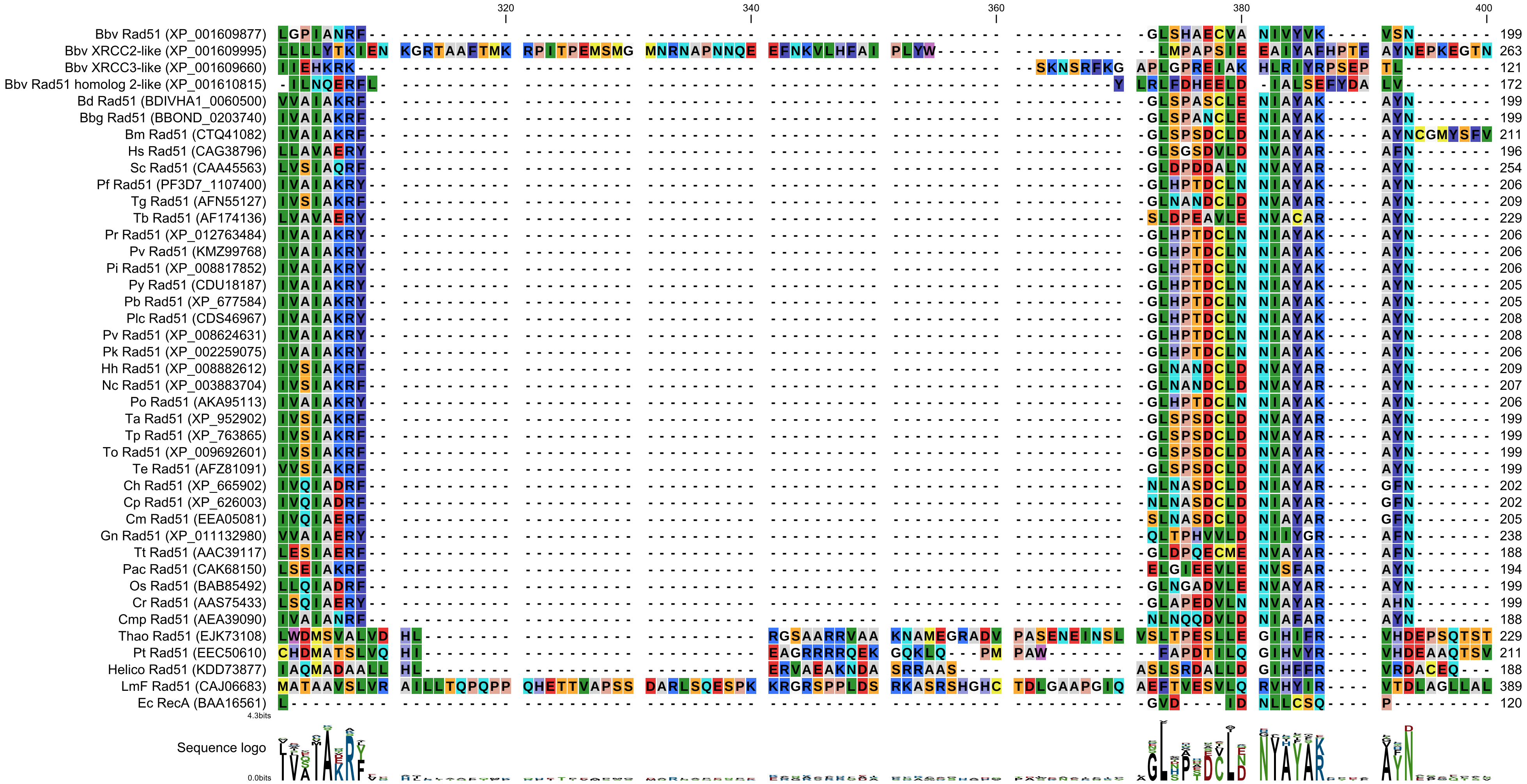
320
340
360
380
400
Bbv Rad51 (XP_001609877)
Bbv XRCC2-like (XP_001609995)
Bbv XRCC3-like (XP_001609660)
Bbv Rad51 homolog 2-like (XP_001610815)
Bd Rad51 (BDIVHA1_0060500)
Bbg Rad51 (BBOND_0203740)
Bm Rad51 (CTQ41082)
Hs Rad51 (CAG38796)
Sc Rad51 (CAA45563)
Pf Rad51 (PF3D7_1107400)
Tg Rad51 (AFN55127)
Tb Rad51 (AF174136)
Pr Rad51 (XP_012763484)
Pv Rad51 (KMZ99768)
Pi Rad51 (XP_008817852)
Py Rad51 (CDU18187)
Pb Rad51 (XP_677584)
Plc Rad51 (CDS46967)
Pv Rad51 (XP_008624631)
Pk Rad51 (XP_002259075)
Hh Rad51 (XP_008882612)
Nc Rad51 (XP_003883704)
Po Rad51 (AKA95113)
Ta Rad51 (XP_952902)
Tp Rad51 (XP_763865)
To Rad51 (XP_009692601)
Te Rad51 (AFZ81091)
Ch Rad51 (XP_665902)
Cp Rad51 (XP_626003)
Cm Rad51 (EEA05081)
Gn Rad51 (XP_011132980)
Tt Rad51 (AAC39117)
Pac Rad51 (CAK68150)
Os Rad51 (BAB85492)
Cr Rad51 (AAS75433)
Cmp Rad51 (AEA39090)
Thao Rad51 (EJK73108)
Pt Rad51 (EEC50610)
Helico Rad51 (KDD73877)
LmF Rad51 (CAJ06683)
Ec RecA (BAA16561)
Sequence logo
4.3bits
0.0bits

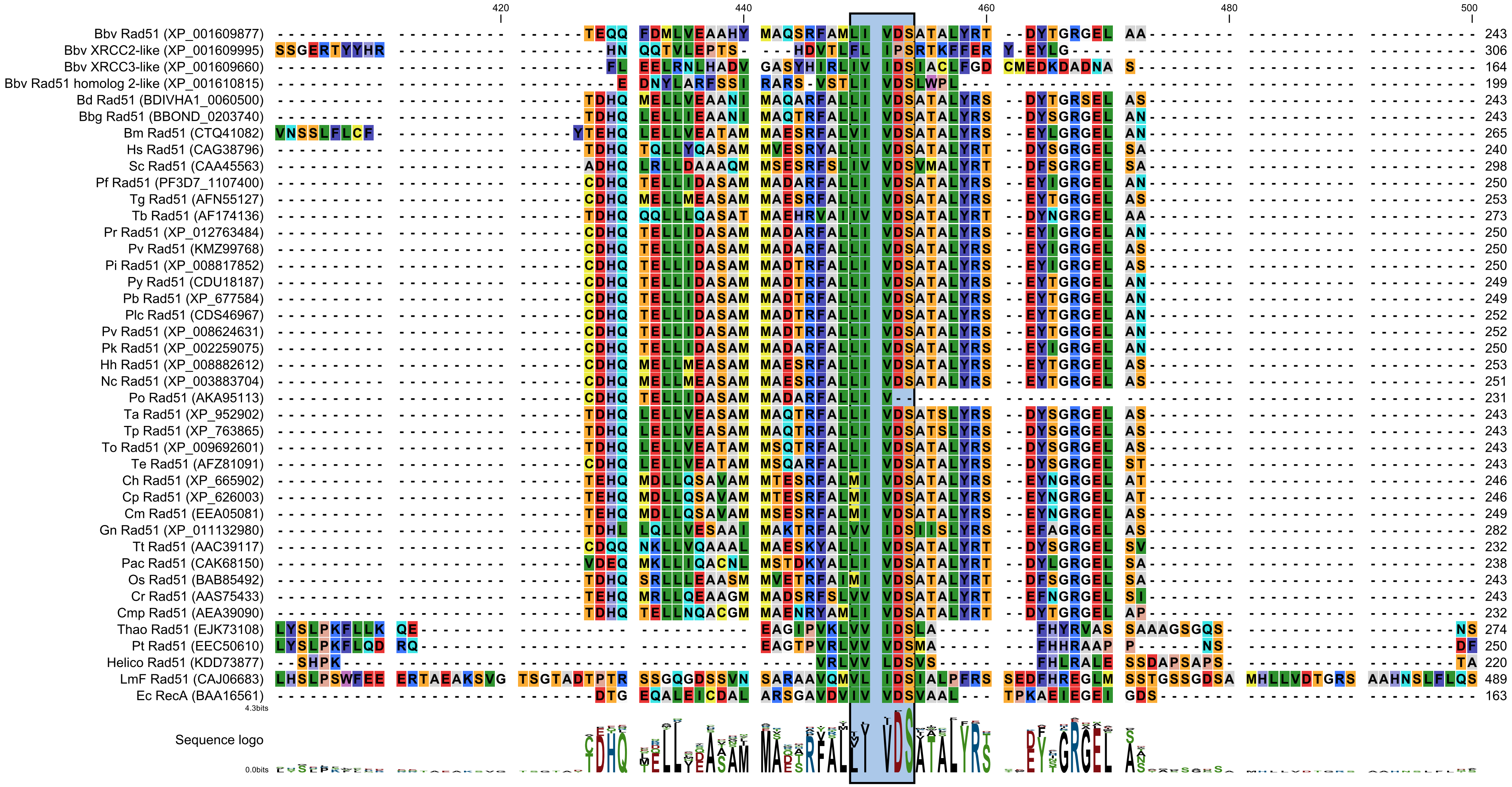
420
440
460
480
500
Bbv Rad51 (XP_001609877)
Bbv XRCC2-like (XP_001609995)
Bbv XRCC3-like (XP_001609660)
Bbv Rad51 homolog 2-like (XP_001610815)
Bd Rad51 (BDIVHA1_0060500)
Bbg Rad51 (BBOND_0203740)
Bm Rad51 (CTQ41082)
Hs Rad51 (CAG38796)
Sc Rad51 (CAA45563)
Pf Rad51 (PF3D7_1107400)
Tg Rad51 (AFN55127)
Tb Rad51 (AF174136)
Pr Rad51 (XP_012763484)
Pv Rad51 (KMZ99768)
Pi Rad51 (XP_008817852)
Py Rad51 (CDU18187)
Pb Rad51 (XP_677584)
Plc Rad51 (CDS46967)
Pv Rad51 (XP_008624631)
Pk Rad51 (XP_002259075)
Hh Rad51 (XP_008882612)
Nc Rad51 (XP_003883704)
Po Rad51 (AKA95113)
Ta Rad51 (XP_952902)
Tp Rad51 (XP_763865)
To Rad51 (XP_009692601)
Te Rad51 (AFZ81091)
Ch Rad51 (XP_665902)
Cp Rad51 (XP_626003)
Cm Rad51 (EEA05081)
Gn Rad51 (XP_011132980)
Tt Rad51 (AAC39117)
Pac Rad51 (CAK68150)
Os Rad51 (BAB85492)
Cr Rad51 (AAS75433)
Cmp Rad51 (AEA39090)
Thao Rad51 (EJK73108)
Pt Rad51 (EEC50610)
Helico Rad51 (KDD73877)
LmF Rad51 (CAJ06683)
Ec RecA (BAA16561)
Sequence logo
4.3bits
0.0bits

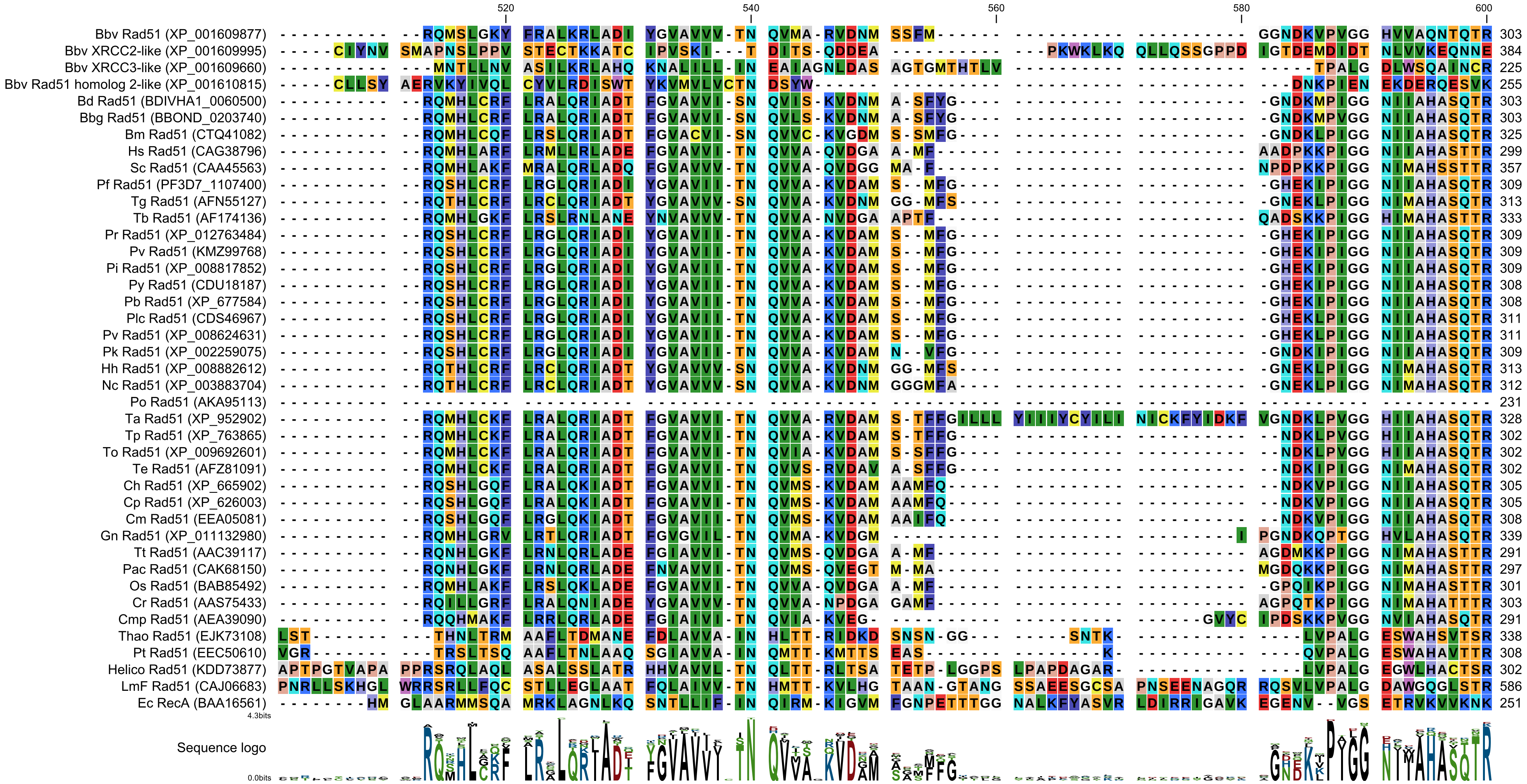
520
540
560
580
600
Bbv Rad51 (XP_001609877)
Bbv XRCC2-like (XP_001609995)
Bbv XRCC3-like (XP_001609660)
Bbv Rad51 homolog 2-like (XP_001610815)
Bd Rad51 (BDIVHA1_0060500)
Bbg Rad51 (BBOND_0203740)
Bm Rad51 (CTQ41082)
Hs Rad51 (CAG38796)
Sc Rad51 (CAA45563)
Pf Rad51 (PF3D7_1107400)
Tg Rad51 (AFN55127)
Tb Rad51 (AF174136)
Pr Rad51 (XP_012763484)
Pv Rad51 (KMZ99768)
Pi Rad51 (XP_008817852)
Py Rad51 (CDU18187)
Pb Rad51 (XP_677584)
Plc Rad51 (CDS46967)
Pv Rad51 (XP_008624631)
Pk Rad51 (XP_002259075)
Hh Rad51 (XP_008882612)
Nc Rad51 (XP_003883704)
Po Rad51 (AKA95113)
Ta Rad51 (XP_952902)
Tp Rad51 (XP_763865)
To Rad51 (XP_009692601)
Te Rad51 (AFZ81091)
Ch Rad51 (XP_665902)
Cp Rad51 (XP_626003)
Cm Rad51 (EEA05081)
Gn Rad51 (XP_011132980)
Tt Rad51 (AAC39117)
Pac Rad51 (CAK68150)
Os Rad51 (BAB85492)
Cr Rad51 (AAS75433)
Cmp Rad51 (AEA39090)
Thao Rad51 (EJK73108)
Pt Rad51 (EEC50610)
Helico Rad51 (KDD73877)
LmF Rad51 (CAJ06683)
Ec RecA (BAA16561)
4.3bits
Sequence logo
0.0bits

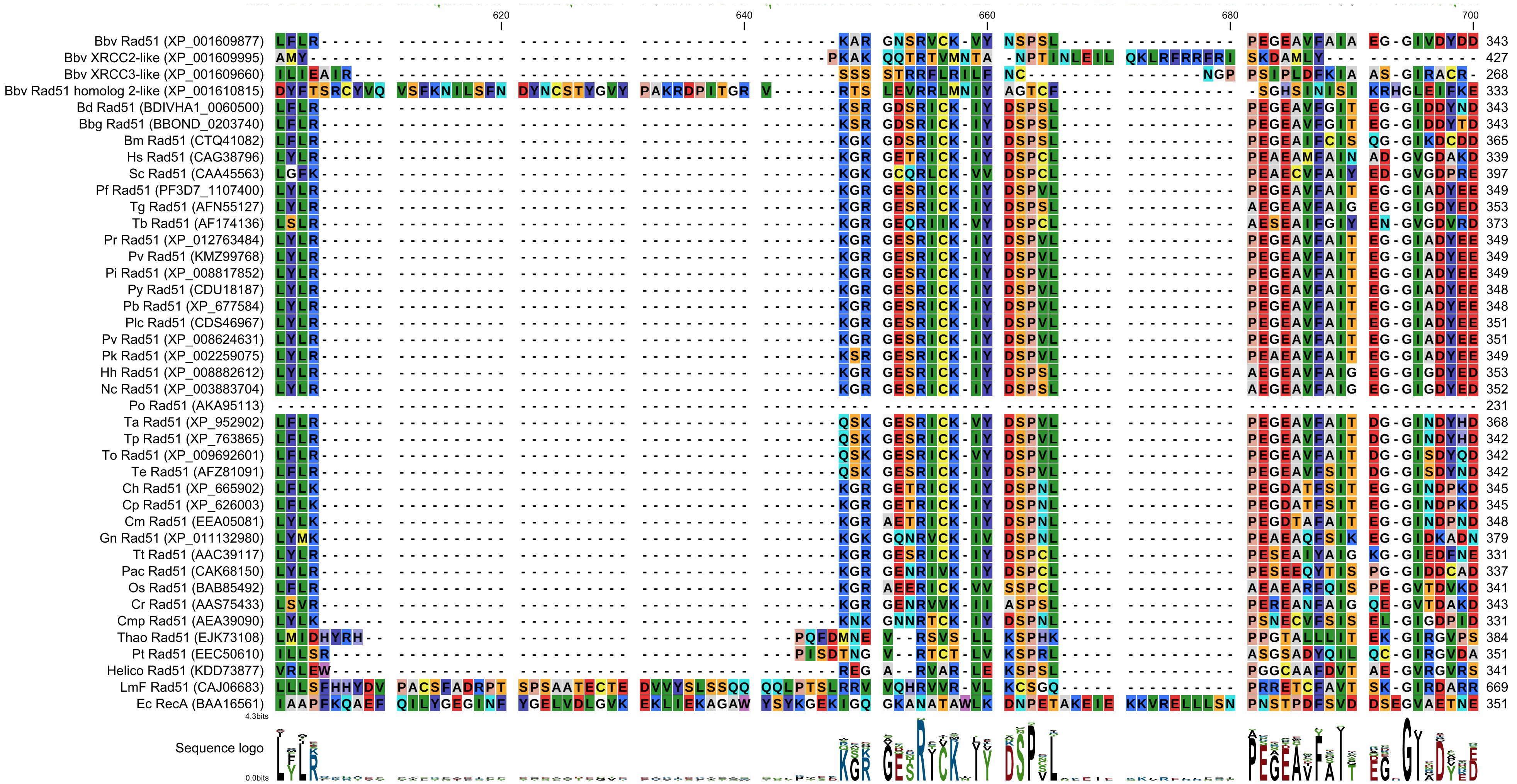
620
640
660
680
700
Bbv Rad51 (XP_001609877) 343
Bbv XRCC2-like (XP_001609995) 427
Bbv XRCC3-like (XP_001609660) 268
Bbv Rad51 homolog 2-like (XP_001610815) 333
Bd Rad51 (BDIVHA1_0060500) 343
Bbg Rad51 (BBOND_0203740) 343
Bm Rad51 (CTQ41082) 365
Hs Rad51 (CAG38796) 339
Sc Rad51 (CAA45563) 397
Pf Rad51 (PF3D7_1107400) 349
Tg Rad51 (AFN55127) 353
Tb Rad51 (AF174136) 373
Pr Rad51 (XP_012763484) 349
Pv Rad51 (KMZ99768) 349
Pi Rad51 (XP_008817852) 349
Py Rad51 (CDU18187) 348
Pb Rad51 (XP_677584) 348
Plc Rad51 (CDS46967) 351
Pv Rad51 (XP_008624631) 351
Pk Rad51 (XP_002259075) 349
Hh Rad51 (XP_008882612) 353
Nc Rad51 (XP_003883704) 352
Po Rad51 (AKA95113) 231
Ta Rad51 (XP_952902) 368
Tp Rad51 (XP_763865) 342
To Rad51 (XP_009692601) 342
Te Rad51 (AFZ81091) 342
Ch Rad51 (XP_665902) 345
Cp Rad51 (XP_626003) 345
Cm Rad51 (EEA05081) 348
Gn Rad51 (XP_011132980) 379
Tt Rad51 (AAC39117) 331
Pac Rad51 (CAK68150) 337
Os Rad51 (BAB85492) 341
Cr Rad51 (AAS75433) 343
Cmp Rad51 (AEA39090) 331
Thao Rad51 (EJK73108) 384
Pt Rad51 (EEC50610) 351
Helico Rad51 (KDD73877) 341
LmF Rad51 (CAJ06683) 669
Ec RecA (BAA16561) 351
4.3bits
Sequence logo
0.0bits

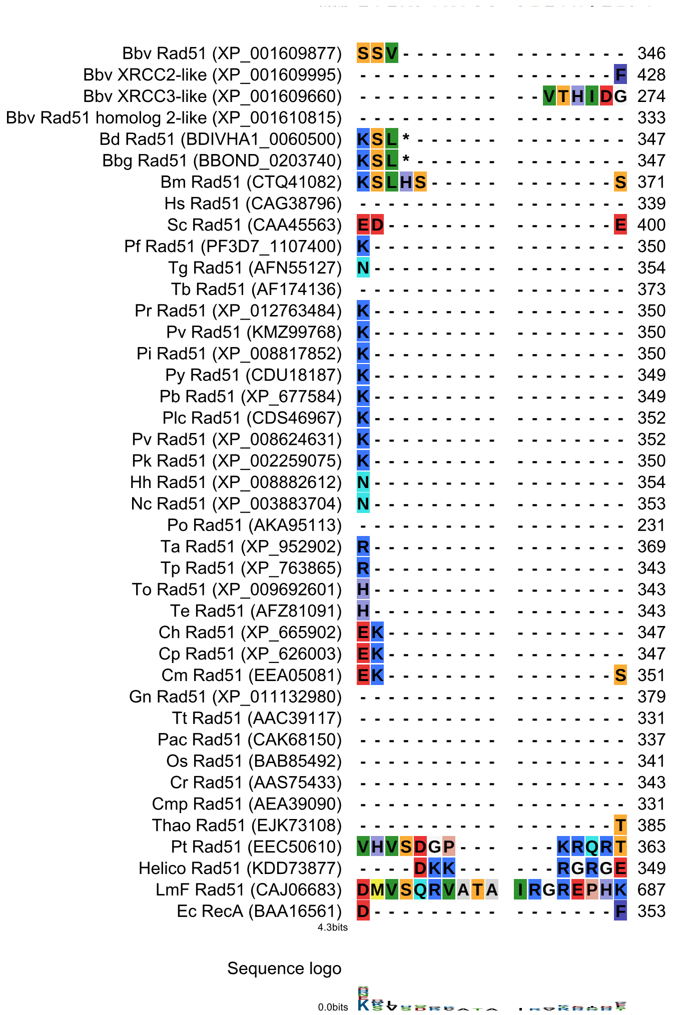
Bbv Rad51 (XP_001609877) SSV 346
Bbv XRCC2-like (XP_001609995) F 428
Bbv XRCC3-like (XP_001609660) VTHIDG 274
Bbv Rad51 homolog 2-like (XP_001610815) 333
Bd Rad51 (BDIVHA1_0060500) KSL* 347
Bbg Rad51 (BBOND_0203740) KSL* 347
Bm Rad51 (CTQ41082) KSLHS S 371
Hs Rad51 (CAG38796) 339
Sc Rad51 (CAA45563) ED E 400
Pf Rad51 (PF3D7_1107400) K 350
Tg Rad51 (AFN55127) N 354
Tb Rad51 (AF174136) 373
Pr Rad51 (XP_012763484) K 350
Pv Rad51 (KMZ99768) K 350
Pi Rad51 (XP_008817852) K 350
Py Rad51 (CDU18187) K 349
Pb Rad51 (XP_677584) K 349
Plc Rad51 (CDS46967) K 352
Pv Rad51 (XP_008624631) K 352
Pk Rad51 (XP_002259075) K 350
Hh Rad51 (XP_008882612) N 354
Nc Rad51 (XP_003883704) N 353
Po Rad51 (AKA95113) 231
Ta Rad51 (XP_952902) R 369
Tp Rad51 (XP_763865) R 343
To Rad51 (XP_009692601) H 343
Te Rad51 (AFZ81091) H 343
Ch Rad51 (XP_665902) EK 347
Cp Rad51 (XP_626003) EK 347
Cm Rad51 (EEA05081) EK S 351
Gn Rad51 (XP_011132980) 379
Tt Rad51 (AAC39117) 331
Pac Rad51 (CAK68150) 337
Os Rad51 (BAB85492) 341
Cr Rad51 (AAS75433) 343
Cmp Rad51 (AEA39090) 331
Thao Rad51 (EJK73108) T 385
Pt Rad51 (EEC50610) VHVSDGP KRQRT 363
Helico Rad51 (KDD73877) DKK RGRGE 349
LmF Rad51 (CAJ06683) DMVSQRVATA IRGREPHK 687
Ec RecA (BAA16561) D F 353
4.3bits
Sequence logo
0.0bits
