# Supplementary figures and images for: Knockout of Babesia bovis rad51 ortholog and its complementation by expression from the BbACc3 artificial chromosome platform
Source: PLoS One. 2019 Aug 6;14(8):e0215882. doi: 10.1371/journal.pone.0215882 (PMC6684078; doi:10.1371/journal.pone.0215882)

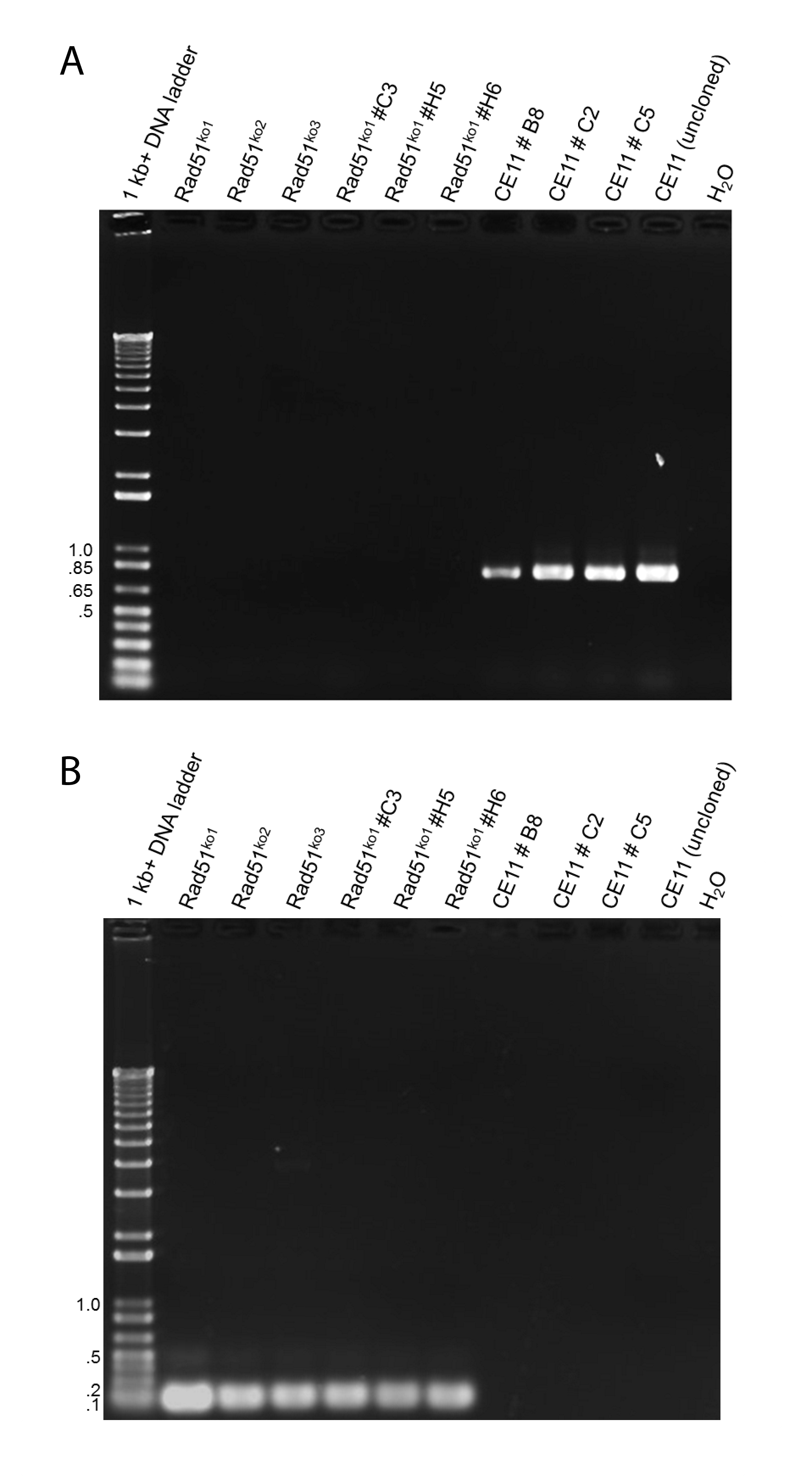

Supplement: S2 Fig — Total RNAs were isolated from B. bovis CE11 subclones B8, C2, and C5, the initial uncloned CE11ΔBbrad51 knockout and three clonal lines derived from it (ko1C3, ko1H5, and ko1H6), and lines CE11Δrad51ko2 and CE11Δrad51ko3. A. cDNAs made with oligo[dT] primer were amplified with primers EAM8 and EAM11 [11, 62] for detection of Bbrad51 transcripts. B. cDNAs were amplified with XW119 and XW121 [11, 62] for detection of gfp-bsd transcripts. Results demonstrate Bbrad51 transcription by wild type lines but not by knockouts. Conversely, gfp-bsd transcripts are present in knockout but not wild type lines. (TIF) [file pone.0215882.s002.tif]

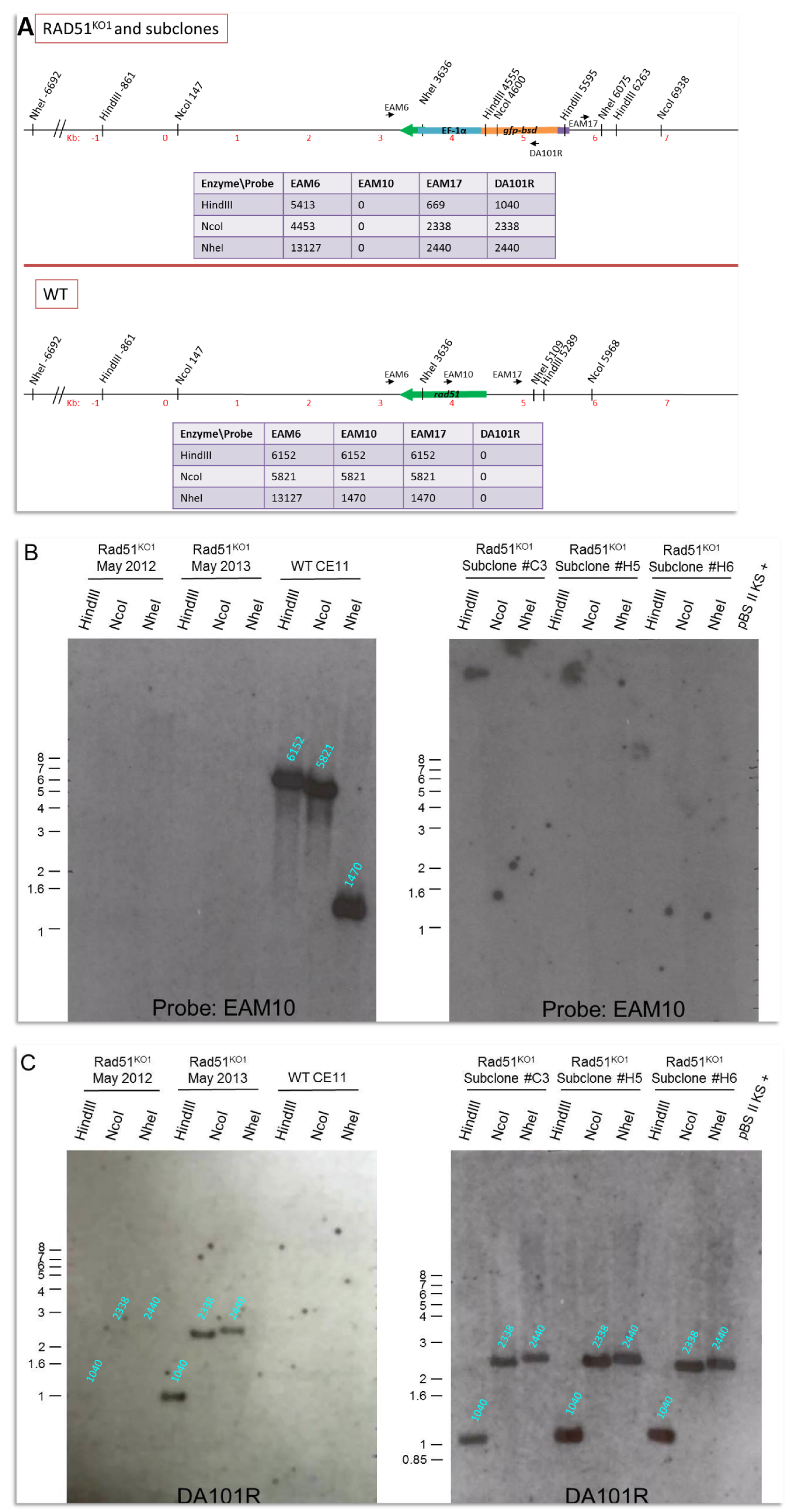

Supplement: S3 Fig — A. Schematic diagram of the Bbrad51 locus in B. bovis CE11 wild type (bottom) and knockout (top) parasites, and the locations of restriction endonuclease and primer binding sites. The tables provide the anticipated sizes (in bp) of specific fragments detected with the indicated probes, based upon the genome sequence. B. Southern blots of B. bovis CE11 wild type, and the initial CE11ΔBbrad51 knockout parasite population both early and late (12 months later) in selection prior to cloning (left panel), after probing with Bbrad51-specific oligonucleotide probe, EAM10. Initial B. bovis CE11ΔBbrad51 clonal lines ko1C3, ko1H5, and ko1H6 are shown in the right panel. C. The same blots shown in panel B are shown after being stripped and re-probed with oligonucleotide DA101R, specific for gfp sequences. The numbers above the bands indicate the sizes of the bands in bp. (TIF) [file pone.0215882.s003.tif]

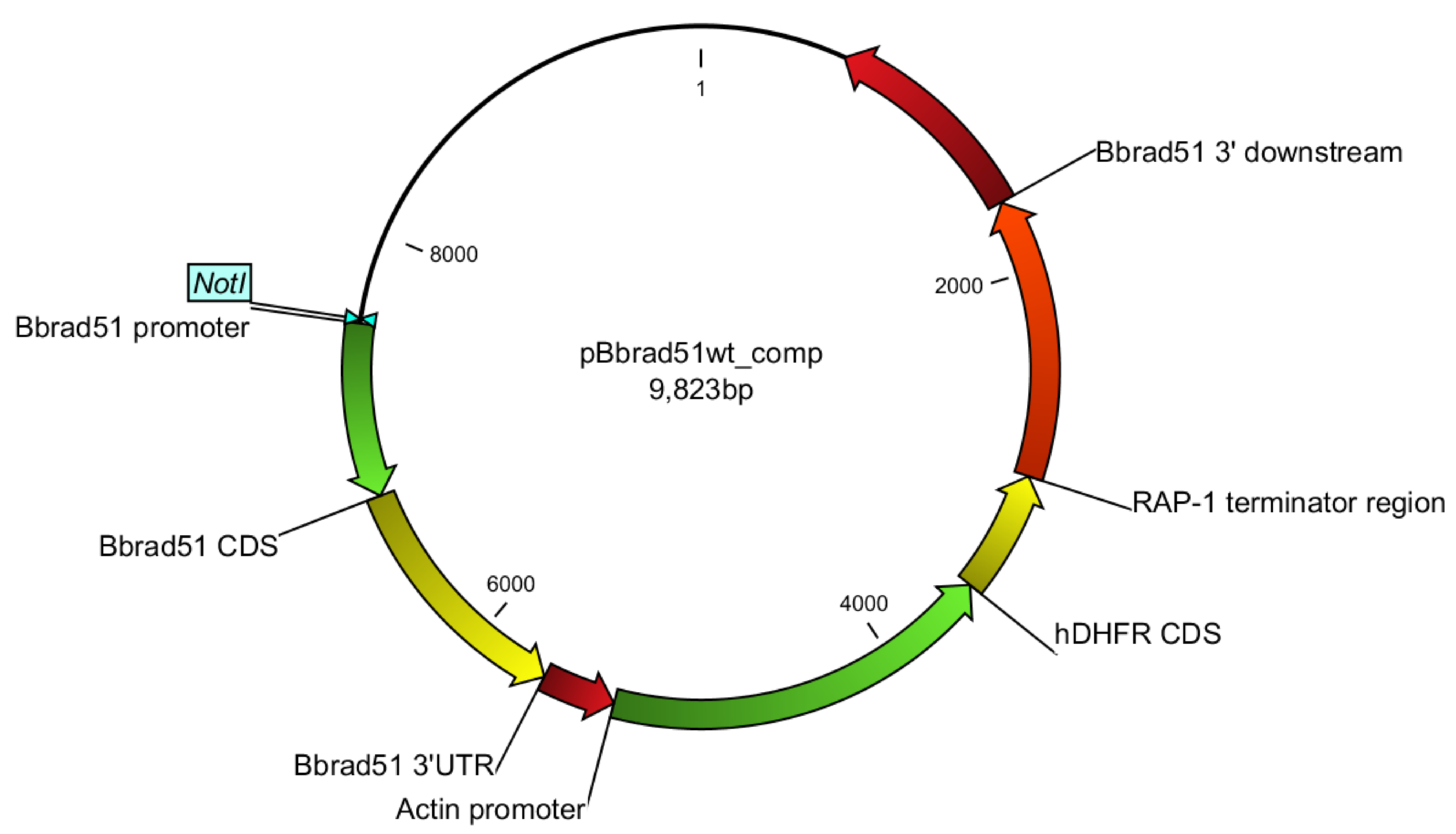

Supplement: S5 Fig — This plasmid was designed to integrate into the genome by double-crossover homologous recombination via sequences upstream and downstream to the Bbrad51 coding sequences. In doing so, the strategy was to recreate the Bbrad51 locus, but with a short 3´-untranslated region for regulation, followed by the hDHFR selection cassette. The plasmid was introduced into parasites after NotI linearization, and selected for growth in the presence of pyrimethamine. This occurred as intended in three of three attempts with wild type parasites, but failed in 11 attempts with B. bovis ko1H5 parasites. (TIF) [file pone.0215882.s005.tif]

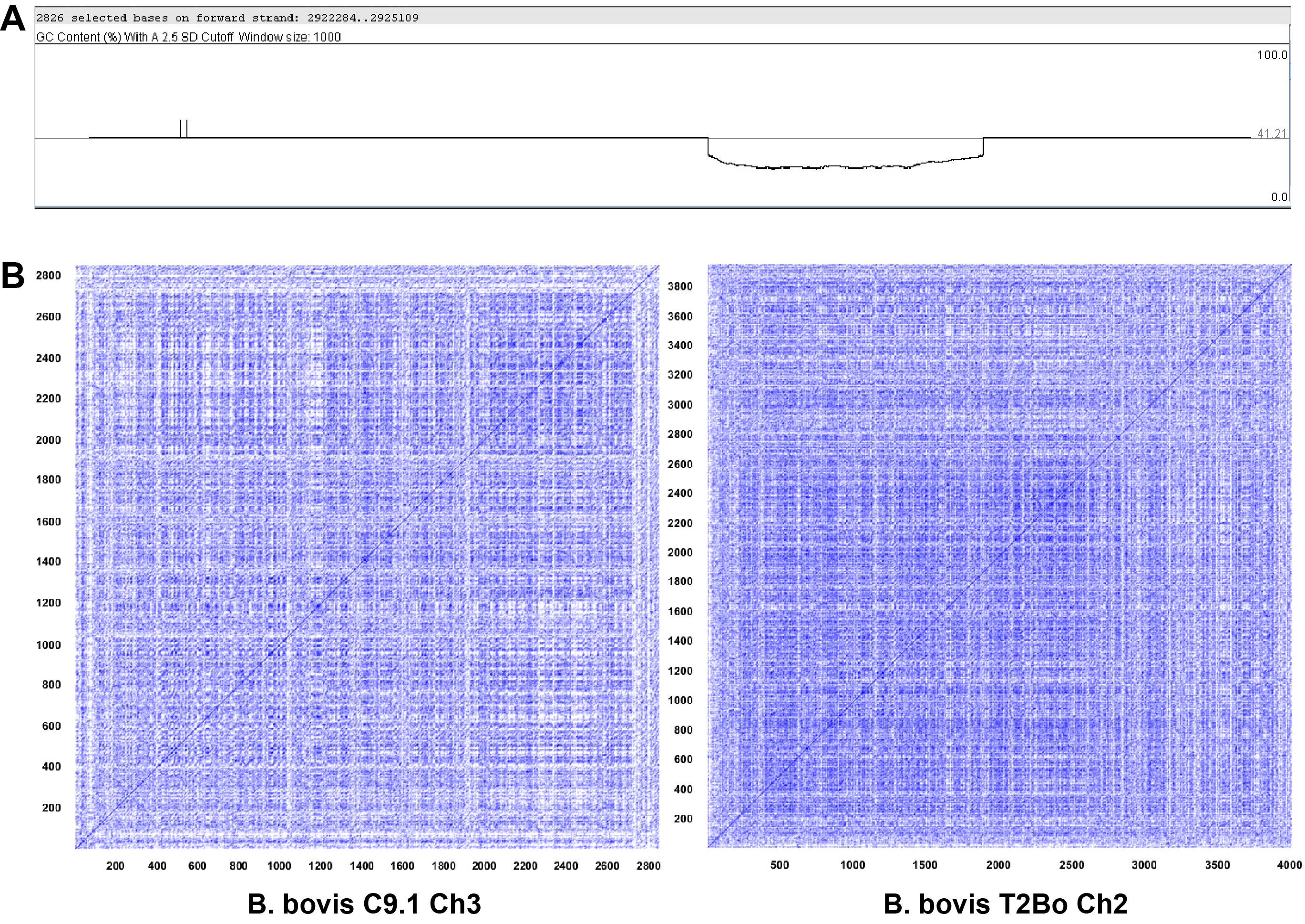

Supplement: S6 Fig — A. B. bovis T2Bo isolate genomic sequences [32] were scanned with the “GC Content (%) with 2.5 SD Cutoff” subroutine of Artemis v. 16.0.0 [76], using a sliding window of 1000 bp. Stretches of sequence > 2.5 standard deviations below the mean G+C content, and ≥ 2 Kbp in length, were identified. Shown is the region of chromosome 3 identified by this strategy. Sequence comprising nucleotides 2922285–2925108 of the B. bovis C9.1 line genome [43], corresponding to nucleotides 29912–32736 of the B. bovis T2Bo isolate chromosome 3 [32], were recovered by PCR and used in the construction of pBbACc3. B. Dot-plots of the putative B. bovis C9.1 line chromosome 3 (left plot) and T2Bo isolate chromosome 2 (right plot) centromeres against themselves. The internal repeat structure of each is apparent from the plots, with the chromosome 2 centromere having a larger major repeat domain. The darkness of spots indicates the degree of similarity, with the dark blue diagonal lines indicating identity. Values within the range from 40–100% identity is shown. When plotted against one another there is no evidence for any specific sequence relationship (not shown). (TIF) [file pone.0215882.s006.tif]

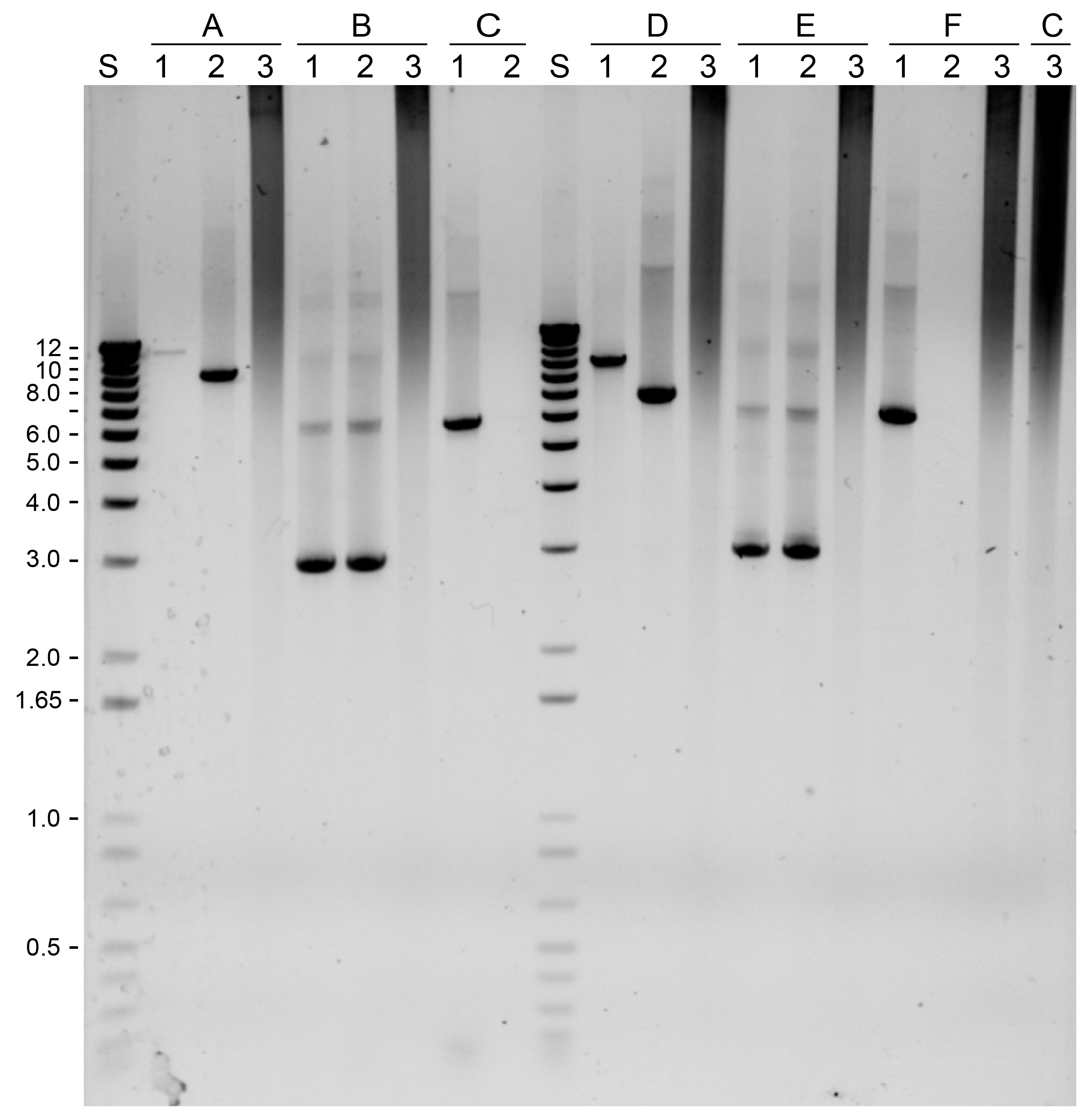

Supplement: S7 Fig — Genomic DNAs were isolated from B. bovis ko1H5, ko1H5/BbACc3, and ko1H5/BbACc3_Bbrad51wt after establishment in in vitro culture. Overlapping internal fragments of the BbACc3 derivatives were amplified by PCR, as well as full-length constructs, minus the telomeric repeat sequences. No evidence for amplification or rearrangements of internal sequences was observed. Lanes are: (S) dsDNA size standards, (1) B. bovis ko1H5/BbACc3_Bbrad51wt gDNA, (2) B. bovis ko1H5/BbACc3 gDNA, and (3) B. bovis ko1H5 gDNA. Primer combinations are indicated above the lanes as: (A) DA291 + DA383, (B) DA291 + DA190, (C) DA291 + DA312, (D) DA291 + DA165, (E) DA383 + DA164, and (F) DA383 + DA313 (“C” samples became split by misplacement of the standard). Expected band sizes were as follows: A1, 11,526 bp; A2, 9285 bp; A3, no amplification (noA); B1, 2989 bp; B2, 2989 bp; B3, noA; C1, 5942 bp; C2, noA; C3, noA; D1, 9107 bp; D2, 6866 bp; D3, noA; E1, 2982 bp; E2, 2982 bp; E3, noA; F1, 6240 bp; F2, noA; F3, noA. Amplification patterns and observed band sizes were consistent with expectation in each instance. (TIF) [file pone.0215882.s007.tif]
