# Supplementary material for: Knockout of Babesia bovis rad51 ortholog and its complementation by expression from the BbACc3 artificial chromosome platform
Source: PLoS One. 2019 Aug 6;14(8):e0215882. doi: 10.1371/journal.pone.0215882 (PMC6684078; doi:10.1371/journal.pone.0215882)
Supplement: S1 Table — (DOCX) [file pone.0215882.s008.docx]

**S1 Table. Putative *B. bovis* orthologs of DNA repair proteins**.

| **Protein used in search** | **Functions/ pathways^1^** | ***B. bovis* protein^2^, Accession #** | **Pfam domains, families, comments^3^** |
| --- | --- | --- | --- |
| Artemis  Q96SD1.2 | 5’ to 3’ exonuclease; NHEJ in V(D)J recombination | *no apparent ortholog found* | No significant similarities found |
| ATM  AAB65827.1 | S/T kinase; DSB sensor and DNA damage checkpoint activator; activates DSB cascade | XP_001612268.1 (4e-33) | Cd00892 PIKKc_ATR (2.69e-105); pfam02260 FATC (8.66e-07); cl21456 periplasmic binding protein (3.57e-03) |
| ATR  CAA70298.1 | S/T kinase; senses persistent ssDNA at stalled replication forks and activates DNA damage checkpoint | XP_001612268.1 (2e-55) | Cd00892 PIKKc_ATR (2.69e-105); pfam02260 FATC (8.66e-07); cl21456 periplasmic binding protein (3.57e-03) |
| BRCA1  AAC37594.1 | DNA-binding RING domain zinc-finger protein; HR and MMR | *no apparent ortholog found* | XP_001612101.1 top hit (4e-07); cd00162 RING (1.85e-11); PLN03208 ubiquitin-protein ligase multi-domain family (1.27e-19) |
| BRCA2  AAB07223.1 | ssDNA-binding; HR repair of dsDNA breaks; displaces RPA | *no apparent ortholog found* | No significant similarities found |
| Bre1  KZV12157.1 | Ubiquitin protein ligase; meiotic DSB formation | *no apparent ortholog found* | XP_001612124.1 top hit (9e-08); cd00162 RING domain |
| DNA ligase IV  P49917.2 | ATP-dependent DNA ligase; NHEJ | *no apparent ortholog found* | XP_001609317.1, DNA Ligase I top hit (4e-37); cd07900; cd07969; pfam04675; PLN03113 (DNA Ligase 1 provisional; 0e+00) |
| ERCC1  NP_973730.1 | DNA excision repair; structure specific endonuclease (with ERCC4); HR, NER, NHEJ | XP_001612291.1  (E= 4e-14) | Cl04283 Rad10 superfamily (E= 1.05e-11); COG5241 Rad10 (E= 1.27e-07); lacks helix-hairpin-helix domain |
| ERCC4  Q92889.3 | Structure specific endonuclease (with ERCC1); HR, NER, NHEJ | XP_001609420.1  (E= 2e-39) | pfam02732 ERCC4 (1.84e-23); TIGR00596 rad1 (3.88e-96) |
| Exo1  Q9UQ84.2 | 5' - 3' exonuclease/double Holliday junction resolution; meiotic recombination | XP_001609909.1  (E= 6e-45) | cd09857 PIN_EXO1 (1.50e-77); cd00901 H3TH_FEN1-like (2.89e-19); TIGR03674 fen arch (4.79e-27) |
| Ku70  *P32807.2* | Binds DSB ends (with Ku80); NHEJ | *no apparent ortholog found* | XP_001609119.1 acid phosphatase top hit (*E= 0.0086*); cl13995; PTZ00422 (glideosome-associated protein 50, provisional 0e+00) |
| Ku80  *Q04437.1*  P13010.3 | Binds DSB ends (with Ku70); NHEJ | *no apparent ortholog found* | XP_001611701.1 hypothetical top hit (*E= 1.6*); pfam00520 ion transport protein |
| Mre11  P49959.3 | 3' to 5' endo- and exonuclease activity; HR, NHEJ, MMEJ | XP_001611854.1  (E= 4e-74) | cd00840 MPP_Mre11_N (6.38e-38); pfam04152 Mre11 DNA-binding (3.62e-30); TIGR00583 Mre11 (2.60e-89) |
| Msh2  P43246.1 | SSA, MMR (with MSH6), large insertion/deletion repair (with MSH3) | XP_001609031.1  (E= 2e-130) | cl21455 P-loop NTPase superfamily (5.98e-110); pfam05192 MutS III (1.79e-19); cl17822 MutS III superfamily (8.07e-06); smart00533 MutSd (2.99e-48) |
| Msh3  P20585.4 | SSA, large insertion/deletion repair (with MSH2) | XP_001609092.1 (2e-66); | pfam01624 MutS I (3.48e-33), pfam05192 MutS III (3.56e-22), pfam00488 MutSV (1.15e-97), COG249 (DNA repair MutS, 3.41e-156); lacks MutS II domain; possible dual homolog with Msh6 |
| Msh6  P52701.2 | SSA; replication; recombination; MMR (with MSH2) | XP_001609092.1  (4e-97) | pfam01624 MutS I (3.48e-33), pfam05192 MutS III (3.56e-22), pfam00488 MutSV (1.15e-97), COG249 (DNA repair MutS, 3.41e-156); lacks MutS II domain; possible dual homolog with Msh3 |
| Mus81  *Q04149.1*  AAH09999.2 | Structure specific endonuclease; mitotic and meiotic HR crossover pathway (with ERCC4) | XP_001609432.1 (*E= 0.004*) | pfam02732 ERCC4 (8.32e-18) |
| p53  P04637.4 | DNA binding; replication checkpoint control, dsDNA damage sensing | *no apparent ortholog found* | No significant similarities found |
| Rad1  P55042.2 | ssDNA endonuclease; mitotic recombination; meiotic MMR; SSA; NER | *no apparent ortholog found* | XP_001611576.1 top hit (E= 3e-16); P-loop NTPase; Rab6-like protein |
| Rad5  P0CQ66.1 | Snf2/ Rad54 family helicase; ubiquitin ligase; DSB repair; error-free post-replication repair; error-free DNA damage tolerance | XP_001611996.1 (2e-61) | pfam00271 Helicase C (3.52e-24); pfam13639 RING zinc finger 1.31e-06); cd00046 DEXDc (3.37e-03); COG0553 SNF2 family superfamily II helicase (2.28e-53); lacks HIRAN domain |
| Rad16  *DAA07233.1* | helicase; transcription-independent NER | XP_001611996.1  (*E= 3e-107*) | pfam00271 Helicase C (3.52e-24); pfam13639 zinc finger RING (1.31e-06); cd00046 DEXDc (3.37e-03); COG0553 HepA (2.28e-53); COG0553 HepA (1.56e-07) |
| Rad18  Q9NS91.2 | Ubiquitin ligase; TLS (error-prone and error-free); error-free post-replication repair | *no apparent ortholog found* | XP_001610168.1 top hit (E= 0.004); pfam13639 zf-RING_2 (1.03e-14), pfam12678 zf-rbx1 (1.93e-10) |
| Rad50  Q92878.1 | tethers DNA with "zinc hook"; HR; SSA | XP_001612121.1  (E= 3e-54) | cd03240 ABC Rad51 (8.35e-29); cl21455 P-loop NTPase superfamily (3.93e-23); cl12013 BAR superfamily 2.38e-04); cl14813 GluZincin superfamily (3.33e-03); TIGR00606 Rad50 (1.15e-77) |
| **Rad51**  ***CAA45563***  **Q06609.1** | **Recombinase; ss- and dsDNA-binding; HR in vegetative growth and meiosis; replication; SSA** | **XP_001609877.1**  **(*E= 2e-103*)**  **(E= 2e-117)** | **cd01123 Rad51/DMC1/radA P-loop NTPase (E= 3.33e-111); PTZ00035 Rad51 (0e+00)** |
| **Rad51 homolog 2-like (Rad51B)**  **NP_002868.1** | **May promote assembly of pre-synaptic Rad51 filaments** | ***no apparent ortholog found*** | XP_001609877.1 top hit (7e-23); PTZ00035 Rad51 protein superfamily (0e+00);  **XP_001610815 converges on Rad51 homolog 2 proteins by Psi-BLAST (5 iterations; 2e-150)** |
| Rad52  P43351.1 | ssDNA binding; facilitates loading of Rad51 onto ssDNA; recombinase assembly; SSA in humans; BIR; inverse repair of DSBs; post-replication repair; telomere maintenance in humans | *no apparent ortholog found* | XP_001609089.1 top hit (E= 4.6); pfam04003 Utp12 (1.46e-14), pfam00444 Ribosomal L36 (5.49e-04) |
| Rad54  CAA66379.1 | helicase; HR; SSA | XP_001610886.1  (E= 8e-178) | pfam00271 Helicase C (4.97e-25); cd00046 DEXDc (1.12e-18); pfam00176 SNF2_N (4.63e-69) |
| Rad55  *DAA11922.1* | Recombinase; SSA; DSB repair; HR in vegetative growth and meiosis | *no apparent ortholog found* | BAN65789.1 top hit (2e-06) is XRCC3-like; cl21455 P-loop NTPase superfamily; PRK09361 radB |
| Rad57  *DAA11852.1* | Recombinase; HR in vegetative growth and meiosis; SSA; DSB repair | *no apparent ortholog found* | XP_001609877.1 (BbRad51) top hit (1e-16); cd01123 Rad51/DMC1/radA (3.33e-111); PTZ00035 Rad51 (0e+00) |
| RPA  P27694.2 | Heterotrimer; binds and stabilizes ssDNA-binding; HR; replication and damage response | XP_001612170.1  (E= 5e-65) | cd04474 RPA1_DBD_A (3.50e-40); pfam16900 REPA_OB_2 (7.90e-35); cl09930 RPA_2b-aaRSs_OBF-like superfamily (8.82e-34) |
| Spo11  *P23179.1*  Q9Y5K1.1 | Topoisomerase complex component; meiosis-specific DNA recombination | *no apparent ortholog found* | no significant similarities found |
| Srs2  *DAA08708.1* | DNA helicase; anti-recombinase; DNA repair; Rad51 nucleoprotein disassembly; meitotic recombination | *no apparent ortholog found* | XP_001610816.1 top hit (E= 0.65); PTZ00341 Ring-infected erythrocyte surface antigen |
| **XRCC2**  **O43543.1** | **X-ray repair cross-complementing protein; recombinase** | ***no apparent ortholog found*** | XP_001609774.1 top hit (E= 4.6); cd00009 AAA+ ATPase superfamily (5.80e-20); pfam08542 Replication factor C C-terminal domain (2.49e-14); cl24691 AAA C-terminal domain (1.58e-05); **XP_00160995 converges on XRCC2 proteins by Psi-BLAST (5 iterations, 2e-122)** |
| **XRCC3**  **O43542.1** | **Rad51C-like; HR; Holliday junction resolution (with Rad21)** | **XP_001609660.1**  **(E= 2e-15)** | **cl21455 P-loop NTPase superfamily (1.82e-26); PRK09361 radB (1.00e-09)** |
| XRCC4  Q13426.2 | DNA ligation (with LIG4); NHEJ; V(D)J recombination | *no apparent ortholog found* | No significant similarities found |

^1^For a comprehensive and authoritative listing of DNA repair protein functions and pathways the reader is referred to the online database REPAIRtoire (<http://repairtoire.genesilico.pl/>).

^2^*B. bovis* proteins were considered orthologs only if, upon reverse Psi-BLAST analysis (typically 3 or 4 iterations), searches converged upon the protein class used to initiate the search. Significance (E) values provided are that of the initial BLAST search that identified the putative *B. bovis* protein, using the human protein as bait. Searches using a *S. cerevisiae* protein as bait are indicated by italics. Iterative reverse Psi-BLAST searches generally yielded E values of 0.0 upon, or even before convergence.

^3^E values provided indicate the significance of match for each listed *B. bovis* protein domain with prototype domains of the Conserved Domain Database and/or Pfam databases.
