# Supplementary material for: Knockout of Babesia bovis rad51 ortholog and its complementation by expression from the BbACc3 artificial chromosome platform
Source: PLoS One. 2019 Aug 6;14(8):e0215882. doi: 10.1371/journal.pone.0215882 (PMC6684078; doi:10.1371/journal.pone.0215882)
Supplement: S2 Table — (DOCX) [file pone.0215882.s009.docx]

**S2 Table. Primers used in this study.**

| **Primer** | **Sequence** |
| --- | --- |
| 3’RACE inner | CGCGGATCCGAATTAATACGACTCACTATAGG |
| 3’RACE outer | GCGAGCACAGAATTAATACGACT |
| 5’RACE inner | CGCGGATCCGAATTAATACGACTCACTATAGG |
| 5’RACE outer | GCTGATGGCGATGAATGAACACTG |
| DA152 | GCGTTTAAACTGTATAGGTGCCACAC |
| DA153 | CGTTTAAACTCGCGATCCAATACAG |
| DA162 | CCGCTCTTGAGACAATAACCCTG |
| DA163 | CCGCACATTTCCCCGAA |
| DA164 | ATGGTTGGTTCGCTAAAC |
| DA165 | TTAATCATTCTTCTCATATACTTCAAA |
| DA170 | GGGATTTTGGTCATGATAGCATAATGAGGATGTTAAG |
| DA171 | TTTGATAATCTCATGAGTTACCCGTATATGTAGTGCACCTAG |
| DA172 | CTATACAGTTTAAACGCCTAAACCCTAAACCCTAAAAC |
| DA173 | ATCGCGAGTTTAAACGCCTAAACCCTAAACCCTAAAAC |
| DA174 | GTCTCAAGAGCGGCCGC\|TGGTGGAGTATTTTGGTAGACAGAG |
| DA175 | GGGAAATGTGCGGCCGCAATGTTCTACTCAAAACATTTTTCG |
| DA189 | ATGTAGGTTGTGATTTTAGATGAC |
| DA190 | GCACCAAAAAAATCATGTGTAAG |
| DA248 | CTAGAACTAGTGGATCCGCAGAACGATAAACAACG |
| DA252 | CGCAATTTGCTTGATAAGCTTGCGCTTTAATCCATTGTATG |
| DA253 | GGAATTCGATATCAAGCTTATCAAGCAAATTGCG |
| DA254 | GGTACTTTCTGGGTCGACCTACGAACGATATGTCAAAGAG |
| DA255 | GACATATCGTTCGTAGGGTCGACCAGAAAGTACCATTATTGGTTTTG |
| DA256 | CGGGCCCCCCCTCGAGGTGTTGCCATCGGTTATTG |
| DA281 | CTTGATATCGAATTCCTGCAGCGCTTTAATCCATTGTATG |
| DA290 | TCCTGTTTTTGCTCACC |
| DA291 | ATCTGTCTATTTCGTTCATCC |
| EAM1 | GAATCTGGCAGCATTACCGA |
| EAM2 | TATAGACTTTGCACACTCTGCTG |
| EAM3 | GCGGAGAAGGGAAATGTCTA |
| EAM4 | TACCTGGTTAGTCACAACTACTGC |
| EAM6 | CATTGATTTAATGTATCGCTACG |
| EAM8 | TTGAGTGTTCTGAGCAACTACA |
| EAM11 | GGATATGTGACCCTGGATAGTA |
| EAM18 | TGAGGTAATGCCATAATACAA |
| EAM42 | CTACGATTAACATGGCAAACCCACGTAATAAATGAGATAAATAA |
| EAM43 | TCTTCTCCTTTGGAGGCCATTTTCGTAAAGTTGCAATAAATTATATC |
| EAM44 | TAATTTATTGCAACTTTACGAAAATGGCCTCCAAAGGAGAAGA |
| EAM45 | GTTATGTGTGGGAGGGCTAATGGAATAACAAGCGCAAGAC |
| EAM46 | TACGTCATTACATAAAGGTGCtGAACTTATGCAATAAGGTTATA |
| EAM47 | TCCAGAATTCCGCAGAACGATAAACAACGCA |
| EAM49 | ATATATTTATTTATCTCATTTATTACGTGGGTTTGCCATGTTAATCGTA |
| EAM50 | CTGCCTCGAGTACAGAGCTATCGTCATAAT |
| EAM51 | TAACGGTACCATGTCTTGCCAAGAGCTGC |
| EAM56 | AGTGCGGCCGCTAGATAATTAGTAATTGTGGTTATATG |
| EAM75 | ATTGAGGTAATGCCATAATACAAAC |
| EAM77 | GTATGCTGGGCATTACCTACC |
| qPCR-Bbrad51-5´F | ACCTATGCCTCGGGACTACAG |
| qPCR-Bbrad51-5´R | GGAAGCGACCTGGGCTATAC |
| qPCR-Bbrad51-3´F | ACGGTGTCGCAGTAGTTGTG |
| qPCR-Bbrad51-3´R | TGCTGTTACCTCTCGCTTTTC |
| qPCR-GAPDH-F | TACACGCCACCACTGCTAAC |
| qPCR-GAPDH-R | GGAATGACCTTGCCTACAGC |
| qPCR-hDHFR-F | ACTCAAGGAACCTCCACAAGG |
| qPCR-hDHFR-R | TGCCACCAACTATCCAGACC |
| qPCR-TPX1-F | TGGTCTGTTCCTCATCGACA |
| qPCR-TPX1-R | AAACTTCACCGTGCTTCTCG |
| XW119 | GCCCTCCCACACATAACCAGAG |
| XW121 | AAATGAGAACAGGGGCATC |
