# Supplementary material for: Knockout of Babesia bovis rad51 ortholog and its complementation by expression from the BbACc3 artificial chromosome platform
Source: PLoS One. 2019 Aug 6;14(8):e0215882. doi: 10.1371/journal.pone.0215882 (PMC6684078; doi:10.1371/journal.pone.0215882)
Supplement: S3 Table — (DOCX) [file pone.0215882.s010.docx]

**S3 Table. Copy numbers of Bb*rad51* sequences present in *B. bovis* genomic DNA preparations.**

| Primer set | Genomic DNA | fM (± s.e.)^1^ | Ratio 5´/3´ ends^2^ |
| --- | --- | --- | --- |
| qPCR-Bbrad51-5´ | *B. bovis* CE11 | 11.64 ± 0.71 | 1.04 |
| qPCR-Bbrad51-3´ | *B. bovis* CE11 | 11.16 ± 0.80 |  |
| qPCR-Bbrad51-5´ | *B. bovis* ^ko1^H5 | n.d. | N/A |
| qPCR-Bbrad51-3´ | *B. bovis* ^ko1^H5 | 5.66 ± 0.14 |  |
| qPCR-Bbrad51-5´ | *B. bovis* ^ko1^H5/BbACc3 | n.d. | N/A |
| qPCR-Bbrad51-3´ | *B. bovis* ^ko1^H5/BbACc3 | 3.05 ± 0.17 |  |
| qPCR-Bbrad51-5´ | *B. bovis* ^ko1^H5/BbACc3_Bb*rad51*wt | 0.94 ± 0.01 | 0.48 |
| qPCR-Bbrad51-3´ | *B. bovis* ^ko1^H5/BbACc3_Bb*rad51*wt | 2.88 ± 0.13 |  |
| qPCR-Bbrad51-5´ | *B. bovis* CE11/pBb*rad51*wt_comp | 7.46 ± 0.22 | 1.09 |
| qPCR-Bbrad51-3´ | *B. bovis* CE11/pBb*rad51*wt_comp | 6.86 ± 0.26 |  |
|  |  |  | Ratio hDHFR/gDNA^3^ |
| qPCR-GAPDH | *B. bovis* CE11 | 9.32 ± 0.55 |  |
| qPCR-TPX-1 | *B. bovis* CE11 | 10.82 ± 2.06 |  |
| qPCR-hDHFR | *B. bovis* CE11 | n.d. | N/A |
| qPCR-GAPDH | *B. bovis* ^ko1^H5 | 5.24 ± 0.11 |  |
| qPCR-TPX-1 | *B. bovis* ^ko1^H5 | 4.90 ± 0.57 |  |
| qPCR-hDHFR | *B. bovis* ^ko1^H5 | n.d. | N/A |
| qPCR-GAPDH | *B. bovis* ^ko1^H5/BbACc3 | 2.67 ± 0.16 |  |
| qPCR-TPX-1 | *B. bovis* ^ko1^H5/BbACc3 | 2.93 ± 0.50 |  |
| qPCR-hDHFR | *B. bovis* ^ko1^H5/BbACc3 | 2.36 ± 0.18 | 0.84 |
| qPCR-GAPDH | *B. bovis* ^ko1^H5/BbACc3_Bb*rad51*wt | 1.81 ± 0.10 |  |
| qPCR-TPX-1 | *B. bovis* ^ko1^H5/BbACc3_Bb*rad51*wt | 1.87 ± 0.06 |  |
| qPCR-hDHFR | *B. bovis* ^ko1^H5/BbACc3_Bb*rad51*wt | 1.00 ± 0.02 | 0.54 |
| qPCR-GAPDH | *B. bovis* CE11/pBb*rad51*wt_comp | 5.80 ± 0.77 |  |
| qPCR-TPX-1 | *B. bovis* CE11/pBb*rad51*wt_comp | 7.36 ± 0.76 |  |
| qPCR-hDHFR | *B. bovis* CE11/pBb*rad51*wt_comp | 7.66 ± 0.66 | 1.16 |

n.d.: Not detected.

N/A: Not applicable.

^1^Standard curves were prepared based upon the fM concentrations of purified cloned plasmid sequences, so the sequences detected in genomic DNA samples are reported the same way.

^2^Ratio of 5´to 3´ ends was calculated by first subtracting the portion of 3´ ends provided by BbACc3_Bb*rad51*wt as determined by the concentration of 5´ ends, then dividing the concentration of BbACc3_Bb*rad51*wt 3´ ends/ concentration of 3´ ends provided by the native chromosomes.

^3^Ratio of hDHFR (representing artificial chromosome) to gDNA was determined as [hDHFR]/mean ([GAPDH] + [TPX-1]). Both GAPDH and TPX-1 are single-copy genes.
